# Supplementary material for: Composite topological structure of domain walls in synthetic antiferromagnets
Source: Sci Rep. 2018 Oct 25;8:15794. doi: 10.1038/s41598-018-33780-6 (PMC6202338; doi:10.1038/s41598-018-33780-6)
Supplement: Supplementary file 1 — Supplementary Information [file 41598_2018_33780_MOESM1_ESM.pdf]

Supplementary Information for "Composite topological structure of domain walls in synthetic antiferromagnets"

A. G. Kolesnikov,[1] V. S. Plotnikov,[1] E. V. Pustovalov,[1] A. S. Samardak,[1,2,3]
L. A. Chebotkevich,[1] A. V. Ognev,[1] and Oleg A. Tretiakov[4,1,*]

[1] *School of Natural Sciences, Far Eastern Federal University, Vladivostok 690950, Russia*
[2] *Center for Spin-Orbitronic Materials, Korea University, Seoul, 02841, Republic of Korea*
[3] *National Research South Ural State University, Chelyabinsk, 454080, Russia*
[4] *Institute for Materials Research, Tohoku University, Sendai 980-8577, Japan*

I. HYSTERESIS LOOPS

The measurement results of the reduced magnetization from the hysteresis loops are shown in Fig. 1. The hysteresis loop is taken at the first antiferromagnetic maximum ($t_{\mathrm{Ru}} = 0.9$ nm). In the film there are only few regions, where the hysteresis is observed. From the value of the remanent magnetization it follows that such regions take up only 5%. Almost complete absence of hysteresis indicates that the reversal occurs mainly due to the rotation of the magnetization. In small fields ($H_{\mathrm{c}} = 60$ Oe), there is a small hysteresis loop (see the upper inset). Also, a small hysteresis is observed in the range of fields from 300 to 900 Oe (see the lower inset). The value of the field, at which there is a maximum disclosure of the hysteresis loop, is denoted by H_{c2}. The measurements show that $H_{\mathrm{c2}} = 0.735 \pm 0.015$ kOe. The saturation field is $H_{\mathrm{s}} = 1.8$ kOe. The hysteresis loops obtained at angles $\phi = 0$ and $\phi = 90^\circ$ differ insignificantly.

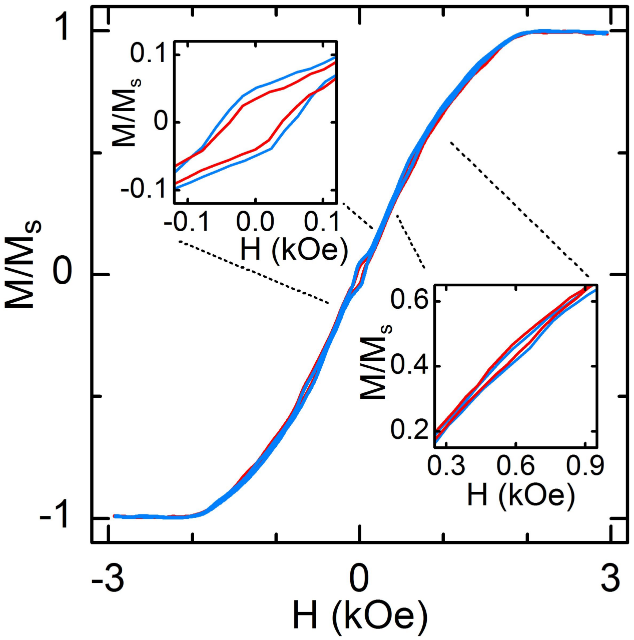

FIG. 1. Magnetic hysteresis loops of Co/Ru(0.9 nm)/Co film: blue line corresponds to H at angle $\phi = 0$, while the red line corresponds to H at angle $\phi = 45^\circ$. Angle $\phi = 0$ corresponds to the easy-axis anisotropy direction in the sample. The insets show the enlarged fragments of the hysteresis loops.

II. ADDITIONAL EXPERIMENTAL DETAILS

Figure 2 shows additional TEM images of the domain structure of Co/Ru(0.9 nm)/Co film obtained by Lorentz microscopy for the fields from 0 to 1.1 kOe.

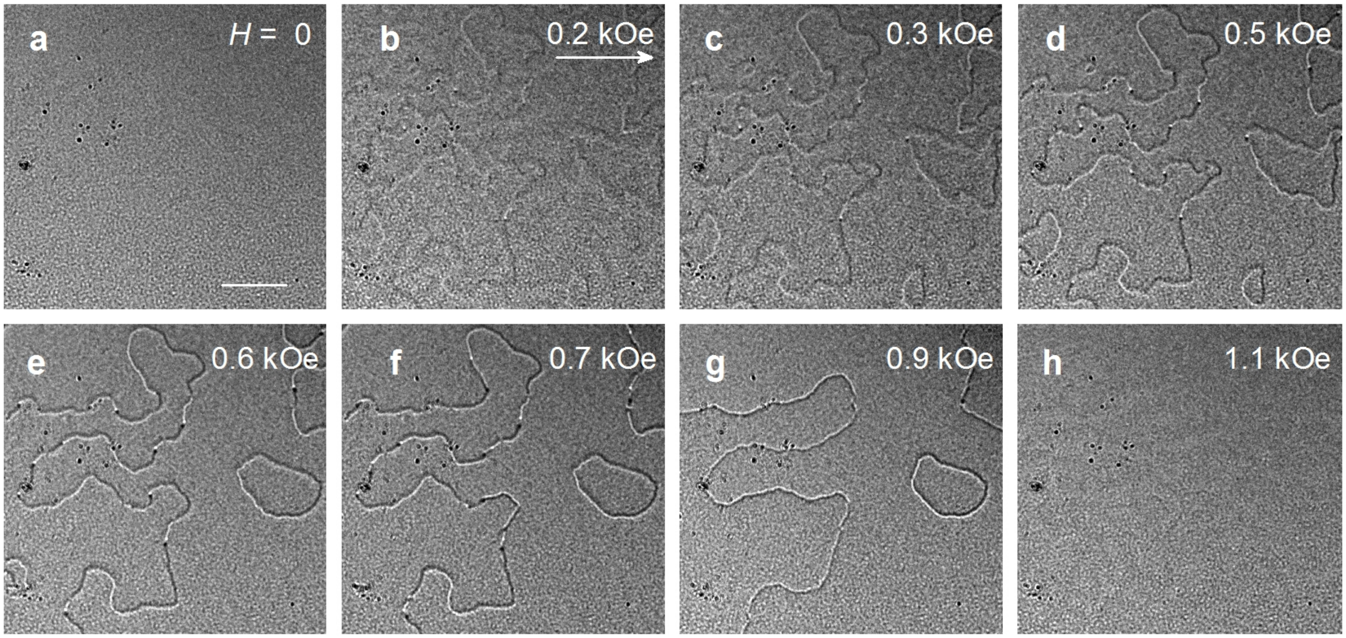

FIG. 2. The images of the domain structure of Co/Ru(0.9 nm)/Co film obtained by Lorentz microscopy for the fields from 0 to 1.1 kOe.

Figure 3 shows additional MFM images of the domain structure for the films Co/Ru(0.9 nm)/Co for the fields in the range from −0.8 to 0.8 kOe.

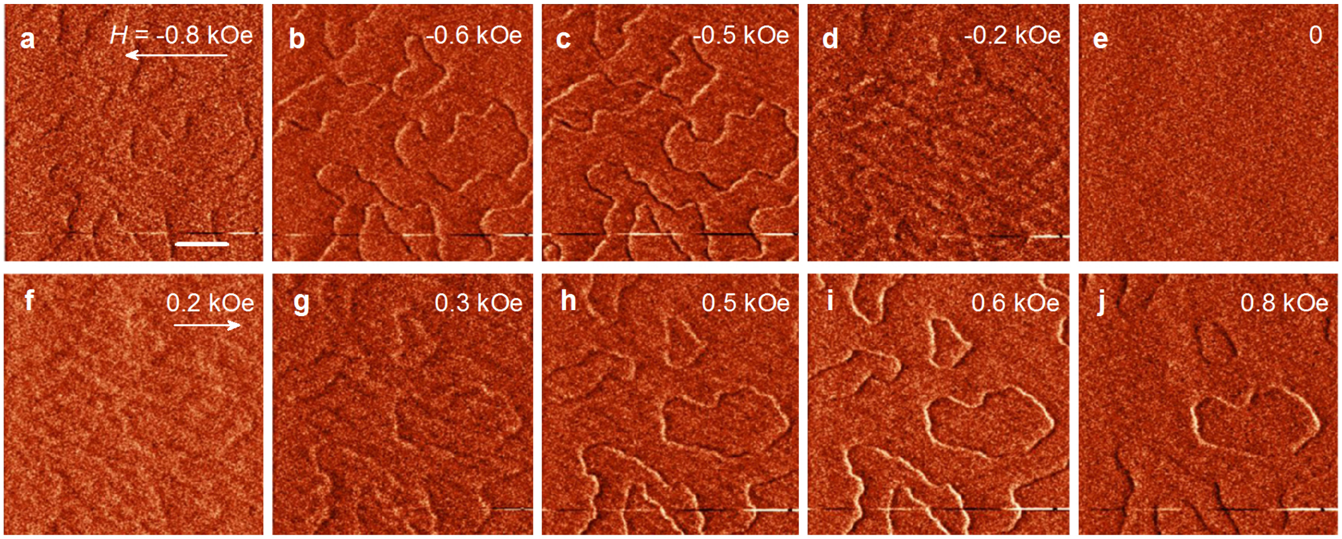

FIG. 3. The MFM images of the domain structure for the films Co/Ru(0.9 nm)/Co are shown for the fields from −0.8 to 0.8 kOe.

SUPPLEMENTARY MOVIES DESCRIPTION

Supplementary Movie 1: Domain wall motion in a synthetic antiferromagnet with increasing magnetic field. The magnetic field is applied perpendicular to the plane (xy-plane). In-plane components of the magnetization at the sites are represented by arrows in a system of 100×120 sites for the time range from $t = 0$ to $t = 3$ ns. The numerical simulations are performed in the film of area $4 \times 4\mu\text{m}^2$ and cell size $4 \times 4 \times 10$ nm^2. The periodic boundary conditions

are applied on the edges. The material parameters of the film were as follows: the saturation magnetization $M_s = 1260$ G, exchange stiffness $A = 3 \times 10^{-6}$ erg/cm, uniaxial anisotropy $K_u = 5 \times 10^4$ erg/cm^3, indirect exchange coupling constant $J_{\rm in} = -1.4$ erg/cm^2, Gilbert damping $\alpha = 0.02$, and magnetic field $B = 320$ Oe. Supplementary Movies 1 and 2: full simulation time is 3 ns. Supplementary Movies 3 and 4: (zoom1) simulation time is 1 ns. Supplementary Movies 5 and 6: (zoom2) full simulation time is 3 ns.

* olegt@imr.tohoku.ac.jp
